# No common factor for illusory percepts, but a link between pareidolia and delusion tendency: A test of predictive coding theory

**DOI:** 10.3389/fpsyg.2022.1067985

**Published:** 2023-01-04

**Authors:** Magdalena Lhotka, Anja Ischebeck, Birgit Helmlinger, Natalia Zaretskaya

**Affiliations:** ^1^Department of Cognitive Psychology and Neuroscience, Institute of Psychology, University of Graz, Graz, Austria; ^2^BioTechMed-Graz, Graz, Austria

**Keywords:** predictive coding, vision, bistable perception, delusions, illusions, hallucinations, Bayesian prior, pareidolia

## Abstract

Predictive coding theory is an influential view of perception and cognition. It proposes that subjective experience of the sensory information results from a comparison between the sensory input and the top-down prediction about this input, the latter being critical for shaping the final perceptual outcome. The theory is able to explain a wide range of phenomena extending from sensory experiences such as visual illusions to complex pathological states such as hallucinations and psychosis. In the current study we aimed at testing the proposed connection between different phenomena explained by the predictive coding theory by measuring the manifestation of top-down predictions at progressing levels of complexity, starting from bistable visual illusions (alternating subjective experience of the same sensory input) and pareidolias (alternative meaningful interpretation of the sensory input) to self-reports of hallucinations and delusional ideations in everyday life. Examining the correlation structure of these measures in 82 adult healthy subjects revealed a positive association between pareidolia proneness and a tendency for delusional ideations, yet without any relationship to bistable illusions. These results show that only a subset of the phenomena that are explained by the predictive coding theory can be attributed to one common underlying factor. Our findings thus support the hierarchical view of predictive processing with independent top-down effects at the sensory and cognitive levels.

## 1. Introduction

Our subjective impression of the outside world results from a complex interplay between the sensory information that our eyes send to our brain on the one hand, and knowledge and experience that we collect throughout our life on the other hand. The influential predictive coding theory aims to explain this interplay by postulating that perception results from an active process of predicting the cause of the current sensory input (Kok and de Lange, 2015; Clark, 2017; Friston, 2018). According to this theory, the brain forms a hypothesis about what caused a certain sensory impression. This hypothesis is then compared with the sensory input by sending a top-down prediction signal. If there is a match, i.e., the hypothesis is able to ‘explain away’ the sensory input, it is equated to our perception. If, however, there is a mismatch between the prediction and the input, termed the “prediction error,” the information about it is resent in a bottom-up fashion for adjusting the prediction. The predictive coding view is often combined with Bayesian inference approach, which considers reliability of the two sources of information when prediction and sensory input are combined (Friston and Kiebel, 2009; Aitchison and Lengyel, 2017). When the top-down expectation (prior) is weak or unreliable, sensory input (evidence) plays a major role in shaping perception. In contrast, when the sensory input is weak or ambiguous, top-down prediction plays a major role in shaping subjective outcome. The predictive coding theory is able to explain a wide range of perceptual and non-perceptual phenomena, ranging from perception of visual illusions in healthy individuals (Hohwy et al., 2008; Kok and de Lange, 2015; Weilnhammer et al., 2017) to pathological states such as hallucinations (Powers et al., 2016) and psychosis (Sterzer et al., 2018), and even such complex phenomena as consciousness (Hohwy and Seth, 2020).

**Figure 1:**
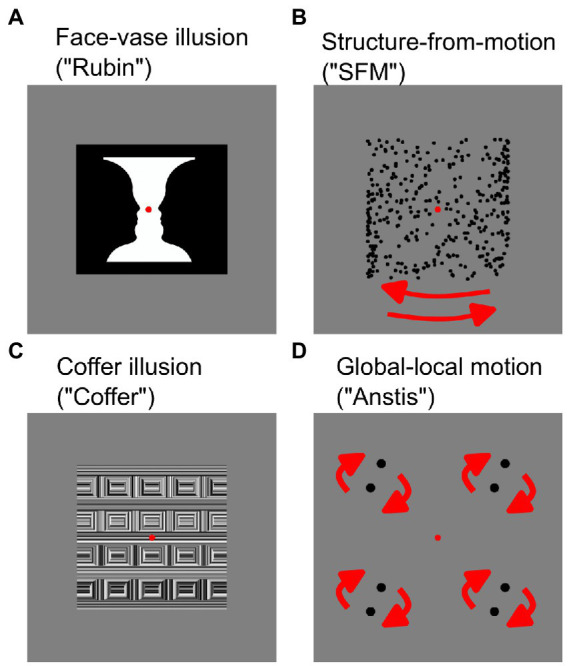
Bistable stimuli used in this study. Static stimuli **(A,C)** and dynamic stimuli **(B,D)**. For dynamic stimuli **(B,D)** movement direction of items is indicated by red arrows.

One type of visual illusions that is often interpreted within the predictive coding framework are the ambiguous (or “bistable”) stimuli. Such stimuli contain visual information that can be interpreted in more than one way. When viewed continuously, such stimuli cause the subjective experience of the observer to alternate between perceiving either one or the other interpretation, with a change in perception occurring every couple of seconds (Long and Toppino, 2004; Brascamp et al., 2018; see also Figures 1A,B). The predictive coding theory yields a straightforward explanation for why the perception changes: after one of the possible interpretations has been selected as the likely cause of the sensory input, the feedback signal about this interpretation is send back to the early processing stages. Since the top-down prediction contains only one of the interpretations, but the sensory input allows for two mutually exclusive ones, the second interpretation is sent forward as the prediction error, which is then used to update the prediction, favoring the second alternative. As long as the sensory input remains the same, there is a constant mismatch between the currently selected interpretation and the ambiguous sensory input, which causes constant prediction updating, and hence a constant change in perception (Weilnhammer et al., 2017; Brascamp et al., 2018).

Among bistable stimuli, one subcategory is particularly intriguing, because one of the possible interpretations appears to be simpler and closer related to the sensory input, while the other represents a more complex illusory impression that is derived from the first, simpler one (Lorenceau and Shiffrar, 1992; Anstis and Kim, 2011; see also Figures 1C,D). Such illusions, termed “asymmetric bistable stimuli” (Grassi et al., 2018), are ideally suited for studying the individual proneness to illusory perception. In contrast to most visual illusions where illusory content is always perceived, here perception alternates between the non-illusory and the illusory interpretation, allowing the quantification of the individual tendency for the latter. The predictive coding explanation of the illusory interpretation is additionally supported by the patterns of brain activity that accompany the more complex illusory interpretation. When a more complex interpretation is perceived, a deactivation of early visual areas is observed, which is interpreted as the top-down prediction matching the sensory input (Murray et al., 2002; Fang et al., 2008; Zaretskaya et al., 2013; Grassi et al., 2018).

Pareidolia is a further form of illusory perception typically associated with predictive coding. Pareidolia is a tendency to recognize familiar forms, most commonly faces, in other meaningful or random objects or patterns (Zhou and Meng, 2020). Examples of pareidolia include recognizing animals in cloud formations, in old tree trunks, or even in radiological images (Alexander et al., 2021). The predictive coding framework offers the most straightforward account of this phenomenon. Specifically, a tendency to recognize familiar items can be seen as manifestation of strong perceptual priors that overweigh the sensory information, especially in situations where the sensory input is weak (Salge et al., 2021). A typical experimental paradigm that induces pareidolia contains stimuli with degraded or ambiguous sensory input and a manipulation that enhances participant’s expectation (i.e., top-down prediction) about the presence of a certain stimulus (Liu et al., 2014; Pajani et al., 2015; Salge et al., 2021). Nevertheless, pareidolias can occur in everyday life even under clear visibility conditions and without a particular expectation of a certain stimulus (Voss et al., 2012).

While the above phenomena are observed in healthy individuals, predictive coding theory is also capable of explaining clinically relevant perceptual and non-perceptual phenomena, such as hallucinations and delusions. Although a mild tendency for both phenomena is encountered in general population, extreme forms may be symptoms of a clinical condition. Hallucinations are sensory impressions that are not related to the actual sensory input. In the context of predictive coding theory, hallucinations are thought to be caused either by a pathologically strong role of predictive mechanisms, or as a failure to accomplish a comparison between prediction and the sensory input and to generate a more accurate prediction error (Powers et al., 2016). Interestingly, links between pareidolia tendency and the presence of visual hallucinations have been reported in some clinical populations that are known to experience hallucinations in the visual modality, such as Parkinson’s disease or dementia with Lewy bodies (Shine et al., 2011; Onofrj et al., 2013). For example, it has been shown that patients suffering from dementia with Lewy bodies exhibit a higher pareidolia proneness compared to controls, both in images of natural scenes and for two-tone noise images (Uchiyama et al., 2012; Yokoi et al., 2014). Similar findings have been demonstrated for Parkinson’s disease patients using ambiguous and unambiguous visual images (Shine et al., 2012). Patients who experienced visual hallucinations were more likely to erroneously identify alternative interpretations in unambiguous images (misperception) and to miss the alternative meaning in the true ambiguous images. In line with the predictive coding explanation of hallucinations as a deficit of top-down influences on perception, recent neuroimaging findings demonstrate specific functional connectivity changes of the frontal areas that are associated with visual hallucinations in Parkinson’s disease (Shine et al., 2015; Kajiyama et al., 2021; Revankar et al., 2021).

In contrast to hallucinations, delusions are non-sensory phenomena and represent aberrant and rigid thoughts and beliefs that are not updated despite the contradicting evidence. As non-sensory phenomena, delusions require a predictive processing explanation at higher non-sensory levels. Crucially, however, dysfunctional sensory predictions are thought to lie at the core of higher-level delusional ideations, both in healthy individuals and as a manifestation of psychotic disease (Sterzer et al., 2018). According to this view, weak top-down sensory predictions (sensory priors) lead to excessive salience of bottom-up sensory events. The excessively salient and overweighed sensory events lead to the formation of aberrant higher-level beliefs that are based on distorted and biased evidence.

In the current study, we tested whether there is indeed a relationship between different perceptual and non-perceptual phenomena that are typically explained by the predictive coding theory in healthy adult individuals. A statistical relationship would indicate not only conceptual similarity, but also a common underlying mechanism. We tested a range of visual perceptual phenomena that contain a dissociation between the sensory input and the actual subjective experience of this input, including two classes of bistable illusions, and two types of pareidolia tasks, one with and one without explicitly induced expectations, and collected self-reports of subjects about their hallucinatory experiences and tendency for delusional ideations. We found a covariation between self-reported tendency for delusional ideations and pareidolia proneness, and a separate, independent covariation between different types of bistable stimuli. We conclude that bistable illusion perception on the one hand, and pareidolia as well as delusion tendency on the other hand, are driven by independent perceptual and cognitive mechanisms.

## 2. Materials and methods

### 2.1. Participants

Eighty two healthy adult volunteers participated in the experiment (mean age: 23.78, SD: 3.29, 55 female). The number of participants was determined using *a priori* power analysis for detecting a correlation at *p* < 0.05 with a power of 80% or more, and considering effects found in previous studies with a similar sample size (Smailes et al., 2020). All participants had normal or corrected-to-normal vision (−0.5 diopters or better) and no history of neurological impairments or psychiatric disorders. Recruiting was performed through the university mailing list as well as through the word of mouth. Since our study exploits individual differences in perception, we deliberately focused on a narrow age group of young healthy adults to reduce variability in perception that is related to age or other factors. Our inclusion criteria as advertised in the study announcements were: age between 18 and 35 years, normal or corrected to normal eye sight, no neurological or psychiatric illnesses, no regular medication intake. Subjects signed a written informed consent prior to participation. They received monetary reimbursement for their time and effort. Psychology students had an option of alternatively receiving course credit. The study was conducted according to the Declaration of Helsinki and was approved by the ethics committee of the University of Graz.

### 2.2. Stimulus and experimental procedures

#### 2.2.1. Vision tests

Prior to the main data collection, we acquired an objective measure of participant’s visual acuity by means of a visual acuity test and a stereoacuity test. Both tests were presented on a Samsung screen (1920 × 1,080 pixels, diagonal display size, 22 inches, vertical refresh rate: 60 Hz, Samsung Group, Seoul, South Korea). First, the Freiburg Computerized Visual Acuity test (FrACT) based on Landolt C’s with 24 trials (Bach, 1996) was conducted with both eyes open, and then separately for each eye at a distance 230 cm from the participant. After this, participants were asked to put on red-blue polarized filters and to perform a random dot V stereotest^1^ with 6 disparity levels, two trials per level in random order. The total stereoacuity score was determined by summing all difficulty levels of correct trials (maximum 42). Visual and stereoacuity data were used to ensure that our results cannot be explained by low-level visual factors.

#### 2.2.2. Bistable illusions

All bistable illusions (Figure 1) were generated using MATLAB R2017b (MathWorks) with Psychtoolbox 3 extensions (Brainard, 1997; Pelli, 1997; Kleiner et al., 2007) on a Linux Ubuntu 18.04 LTS computer and presented using a linearized ASUS VG248QE LCD gaming monitor (1920 × 1,080, diagonal display size: 24 inches, vertical refresh rate: 60 Hz, ASUSTek Computer Inc., Taipei, Taiwan). The screen (53.1 cm width × 29.9 cm height) was placed at a distance of 65 cm, subtending 44.44 × 25.91° in visual angle units and had a maximum luminance of 90.40 cd/m^2^.

All bistable illusions elicited two different interpretations while the physical input remained the same. In two of the illusions (Rubin Face-Vase illusion, Structure-from-Motion stimulus), the two perceptual alternatives were similar in content and complexity, making these illusions symmetric. The other two illusions (Coffer illusion, global–local motion illusion) were asymmetric, with one perceptual interpretation being simpler and the other more complex and illusory (Grassi et al., 2018). Illusions were selected such that there was one static (Figures 1A,C) and one moving dynamic (Figures 1B,D) illusion in each category. Every bistable illusion was presented on a gray background (0.5 of full luminance) with a red fixation dot (0.28° in diameter) in the center of the screen.

In the Rubin’s Face-Vase illusion (Figure 1A), participants were presented with the ambiguous vase-face image (Rubin, 1915). In this image (6.94 × 6.94°), either two face profiles in black facing each other or a white vase could be perceived. In the Coffer Illusion (Figure 1C) the participants were presented with an image (6.94 × 6.94°) of what initially looks like a grid of squares (default percept). Upon longer observation 16 circles (alternative percept) could appear in the image (Norcia, 2006). The dynamic structure-from-motion illusion (“SFM,” Figure 1B) was produced by 350 black dots (dot diameter: 0.16°) that were randomly placed around the fixation point forming a cylinder (cylinder width and height 6.58 × 6.67°). The dots moved horizontally in opposite directions at a speed of 0.56°/s creating the effect of a 3D cylinder structure. Participants perceived the cylinder as rotating to the right or to the left. Finally, in the bistable global–local motion illusion (“Anstis,” Figure 1D) four pairs of black dots (0.42° dot diameter, 1.23° center-to-center distance between two dots in a pair) were arranged in a square (side length: 5.89°). The pairs were rotating in circular motion (0.5 revolutions/s) leading to perception of either four pairs of dots moving locally (default percept) or of two large squares rotating on top of each other (Anstis and Kim, 2011). Short videos of the dynamic stimuli are available as Supplementary material.

Subjects were seated in a chair in front of the monitor with their head in a chin rest to minimize head movement. They were asked to view the stimuli and indicate their perception using the left and right arrow keys on a SteelSeries APEX M800 high-precision mechanical gaming keyboard (SteelSeries ApS, Copenhagen, Denmark). Participants had to keep pressing the key as long as the corresponding percept was experienced, only pressing no key if they perceived both at the same time or were unsure of what they saw. The left arrow was used for faces, leftward cylinder rotation, squares in the Coffer illusion and local motion in the Anstis illusion. The right arrow was used for vase, rightward cylinder rotation, circles in the Coffer illusion and global illusory squares in the Anstis illusion. Before each illusion the participants had a chance to familiarize themselves with the stimulus and to practice.

The four illusions were presented in randomized order. Each illusion was presented four times for 120 s with a 20 s break in between, making the total viewing time of each illusion 8 min long. After each bistable illusion block participants were given the possibility of a self-determined break.

#### 2.2.3. Pareidolia tasks

Either following or preceding the bistable illusion block (in a counterbalanced order) subjects were presented with two different pareidolia tasks (Noise Pareidolia task, Picture Pareidolia task). Both tasks were generated using PsychoPy3 version 2021.1.4 (Peirce et al., 2019) and presented using the same setup in the order that was counterbalanced across subjects.

**Figure 2:**
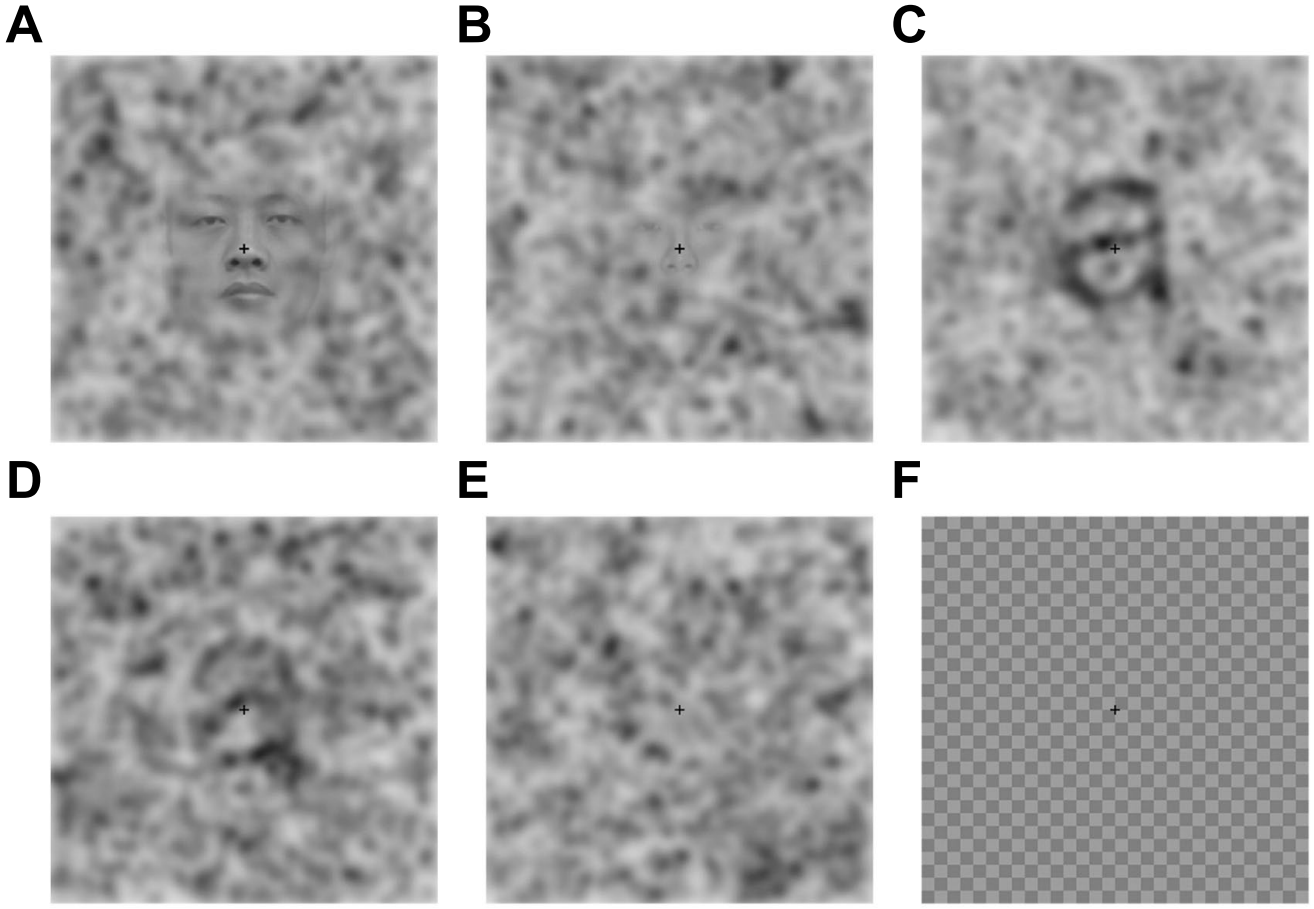
Example stimuli for the Noise Pareidolia face **(A,B)** and letter **(C,D)** detection task as well as pure noise **(E)** and a checkerboard mask **(F)**. Reproduced with permission without changes from Liu et al. (Liu et al. 2014).

*Noise Pareidolia task.* The Noise Pareidolia task aimed at measuring the tendency of participants to perceive expected meaningful items in pure noise and followed the procedure described previously by Liu et al. (2014) using identical stimulation material, but a modified experimental paradigm (for details see below). The stimuli consisted of either faces or letters embedded in noise or of pure noise, yielding 6 experimental conditions: easy-to-detect faces, hard-to-detect faces, pure noise with face expectation, easy-to-detect letters, hard-to-detect letters and pure-noise with letter expectation (Figure 2). The pure-noise images were produced by randomly combining and uniformly spacing bivariate Gaussian blobs with different standard deviations. The same noise images were used in the face and letter task. The easy-to-detect faces and hard-to-detect faces were created from 20 grayscale face photographs (male and female). The faces in the photographs showed a front view and held a neutral face expression. Each face was placed in the center of the image. The face-noise images were created by blending a face photo with a pure-noise image. The letter-noise images consisted of nine Arial Roman/English letter images (a, s, c, e, m, n, o, r, u) and were created by placing a black, printed letter in the center of an image. Identical to the face-noise images, the letter-noise images were created by blending a letter image with a pure-noise image. A checkerboard image was used to neutralize any aftereffects of the images after each trial. For a detailed description of the stimuli see Liu et al. (2014).

Face and letter detection tasks were presented separately in a randomized and counterbalanced order. Each detection task consisted of three blocks and always started with the easy block where 20 easy-to-detect pictures and 20 pure noise images were presented in a randomized order. The next block contained 20 hard-to-detect images and 20 pure-noise images, and the last block included 40 pure noise images. The difficulty, type of stimulus to detect and the instructions were shown to the participants before each block. Each trial started with a fixation cross presented at the center of the screen for 200 ms followed by the stimuli (easy-to-detect, hard-to-detect, noise) for 150 ms, followed by a checkerboard image with a fixation crosshair for 200 ms. Afterwards participants were prompted to give their answer within 3 s by pressing a button.

Participants were asked to press the right arrow on the keyboard if they saw a face/letter in the image and the left arrow if they did not. They were informed that the difficulty of the task would increase from the first to the third block, and that the third block would be the most difficult one. Participants were not informed about the exact percentage of face-containing trials in the most difficult block. At the beginning of each condition participants were presented with five example trials consisting of easy-to-detect, hard-to-detect and pure noise images to familiarize them with the task. The progression from easy to pure noise blocks was intended to induce an expectation of a face/letter in the pure noise blocks. The rate of false positives (i.e., faces or letters identified in pure noise images) in the pure noise block was measured.

**Figure 3:**
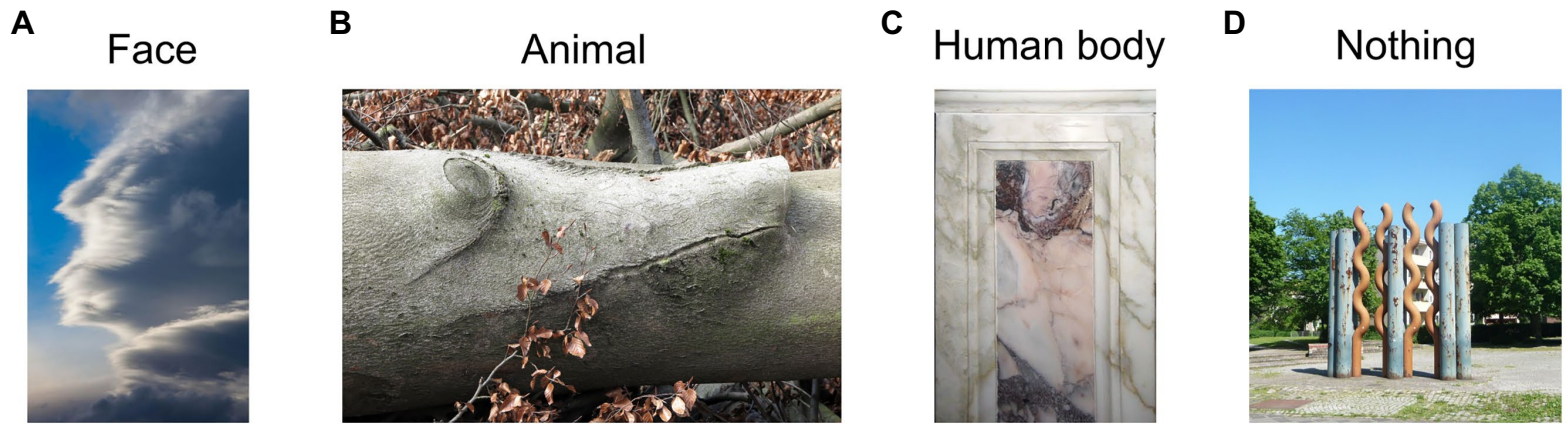
Example picture pareidolia stimuli. Reproduced unchanged from Wikimedia Commons. **(A)** Photograph by Frank Kovalchek. 2012. Wikimedia.org. https://commons.wikimedia.org/wiki/File:Interesting_cloud_-_it_looks_like_the_face_of_a_superhero_(8352871256).jpg CC BY 2.0. **(B)** Photograph by Rolf Brecher. 2018. Wikimedia.org. https://commons.wikimedia.org/wiki/File:Gemeines_Baumschwein_(40153933314).jpg CC BY 2.0. **(C)** Photograph by Fabiolinux25. 2009. Wikimedia.org. https://commons.wikimedia.org/wiki/File:Pareidolia_-_Chiesa_Madre_di_Nereto_-_Teramo,_Italy.jpg Public domain image. **(D)** Photograph by Immanuel Giel. 2013. Wikimedia.org. https://commons.wikimedia.org/wiki/File:Rostiges_Metall_%3D_moderne_Kunst_-_panoramio_(1).jpg CC BY 3.0.

*Picture pareidolia task.* The picture pareidolia task was aimed at determining the participant’s tendency to produce a more complex interpretation of the sensory input. It was designed by the authors and consisted of color photographs of natural scenes with three types of context (woods, clouds and man-made objects) containing either a face, an animal or a human-like body (Figure 3). We deliberately included several object categories and not just faces to make sure we are not investigating abilities related to face recognition, but a general ability to interpret visual information in a meaningful way. An object was hidden in 75% of the images of each context. Each object type was present at least twice in each context. The cloud context contained 3 animals, 3 human-like faces and 2 human-like bodies. The man-made context contained 2 animals, 3 human-like faces and 3 human-like bodies. The wood context contained 3 animals, 2 human-like faces and 3 human-like bodies. This yielded an equal number of images per object category. The images were identified by a web search (primarily on www.commons.wikimedia.org) and were preselected from an initial larger set of images (96) based on a short pilot online experiment with an independent group of participants (*N* = 10). The pilot study aimed to assure that the participants do not indicate any pareidolia in images intended for the “pareidolia-absent” image category but are able to identify the respective objects in the “pareidolia-present” image category. The pilot experiment led to a selection of 39 images in total. Three of these images were used for the practice trial and the remaining 36 images were made up of 24 “pareidolia-present” (8 images per context) and 12 “pareidolia-absent” (4 images per context) images.

Each trial started with a 1-s fixation cross, after which a picture was presented, and participants had 10 s to answer. Participants were instructed to view the pictures and use the mouse to left-click at the center of the object they saw if they saw an animal, a face or human-like body in the photo. If they did not see any figure in the picture they were asked to right-click into the center of the picture. The task started with a practice trial made up of three pictures. In this experiment we were primarily interested in the proportion of correctly identified objects (hit rate).

#### 2.2.4. Questionnaires

The main experimental part was followed by 3 self-rating scales that measure hallucination and delusion proneness. An additional fourth questionnaire that assessed mindfulness (Baer et al., 2004) was also presented to the first 60 tested participants. This data was collected to address an entirely different research question and was therefore not a focus of the current study. The questionnaires were presented electronically using LimeSurvey^2^ using the same setup as the stimuli. Participants were required to use the mouse for answering the questions. The order of the questionnaires was randomized and counterbalanced across individuals. Main quantitative information on the questionnaires is presented in the Supplementary Table 1.

*Cardiff Anomalous Perceptions Scale (CAPS)* is a self-report scale that measures perceptual anomalies (Bell et al., 2006). The 32 items could be answered by the participants with “yes” or “no.” For each item being answered with a “yes” participants were required to rate this item on 5-point subscales on intrusiveness, frequency, and distress. An example item would be: “Do you ever see shapes, lights or colors even though there is nothing really there?” The translation of the CAPS into German was performed using the back-translation procedure (Brislin, 1970; Sperber, 2004). The initial translation was performed by the first author. Afterwards a colleague with a very good to excellent command of English translated the German version back to English. Discrepancies between the original version and the retranslated version were solved consensually.

*Launay-Slade-Hallucinations Scale – R (LSHS-R)* is a self-report questionnaire assessing hallucination proneness in healthy individuals (Launay and Slade, 1981; Bentall and Slade, 1985). Participants were asked to rate each of the 12 items on a 5-point scale from “certainly does not apply to me” to “certainly applies to me.” An example item would be: “In the past, I have had the experience of hearing a person’s voice and then found that no-one was there.” The existing German adaptation of the questionnaire was used (Lincoln et al., 2009).

Peters et al. (1999) *Delusions Inventory (PDI)* measures delusional ideation in the general population. It contains a total of 40 items with dichotomous response format (yes/no). For each item being answered with a “yes” participants were required to rate this item on 5-point subscales on distress, preoccupation, and conviction. An example item would be: “Do you ever feel as if someone is deliberately trying to harm you?” The delusions inventory was added to determine to which extent abnormal perceptual effects quantified with CAPS and LSHS-R are restricted to the perceptual domain or are related to higher-level delusional tendencies. The existing German adaptation of PDI was used (Lincoln et al., 2009).

### 2.3. Data analysis

#### 2.3.1. Subject-level analysis

Subject-level analysis was performed using custom scripts written in GNU Octave version 5.2.0.

*Bistable perception task.* Subject’s button presses during bistable perception blocks were used to determine duration of each perceptual phase. Because the distribution of individual-level dominance durations deviates from the normal distribution (Levelt, 1967; Brascamp et al., 2005), the geometric mean (defined as the n^th^ root of the product of n values) instead of the arithmetic mean was used as a measure of average duration for each illusion of each subject. Additionally, for the asymmetric bistable stimuli we quantified the tendency to perceive the alternative interpretation (“global” for the Anstis stimulus, “circles” for the Coffer illusion), defined as the total time the alternative interpretation was perceived divided by the total time both percepts were perceived.

*Pareidolia tasks*. For the Noise Pareidolia task, in which we measured subject’s tendency to perceive expected stimuli in pure noise, subject’s reports for “stimulus present” in the noise-only blocks were summed for the face and letter task and divided by the total number of noise-only trials, yielding one false alarm rate value per subject. For the Picture Pareidolia task, where we measured subjects’ tendency to overinterpret the content that already had its standard meaning in the absence of explicitly induced expectations, subjects’ correctly identified hidden items were summed over all contexts and all object types and divided by the total number of trials, yielding one hit rate value per subject.

#### 2.3.2. Principal component analysis and pairwise correlations

The group-level analysis was performed in RStudio (22.07.1 + 554). The subject-level analysis described above yielded a multidimensional dataset with 11 values per subject: dominance durations for each of the four bistable illusions (Rubin’s Face-Vase Illusion, Structure-From-Motion Stimulus, Coffer Illusion, Anstis global–local illusion), complex percept predominance for each of the two asymmetric bistable illusions, false alarm rate in the pure noise blocks of the Noise Pareidolia task, hit rate for the Picture Pareidolia task and the scores of the three questionnaires (LSHS-R, CAPS and PDI). These 11 values were used to perform the principal component analysis with *prcomp* function. Variables were standardized prior to PCA (i.e., subtracting the mean and dividing by the standard deviation of each variable). The number of extracted components was chosen based on Horn’s parallel analysis with 10,000 iterations (Horn, 1965) as implemented in *paran* library. To test which of the dependent variables covary, we conducted a varimax rotation on extracted components (rotating the axes of principle components to maximize the separation of individual variables across components). Finally, since most variables were not normally distributed (Supplementary Figure 1), the correlation structure between individual variables was examined using Spearman’s correlation coefficient.

## 3. Results

**Figure 4:**
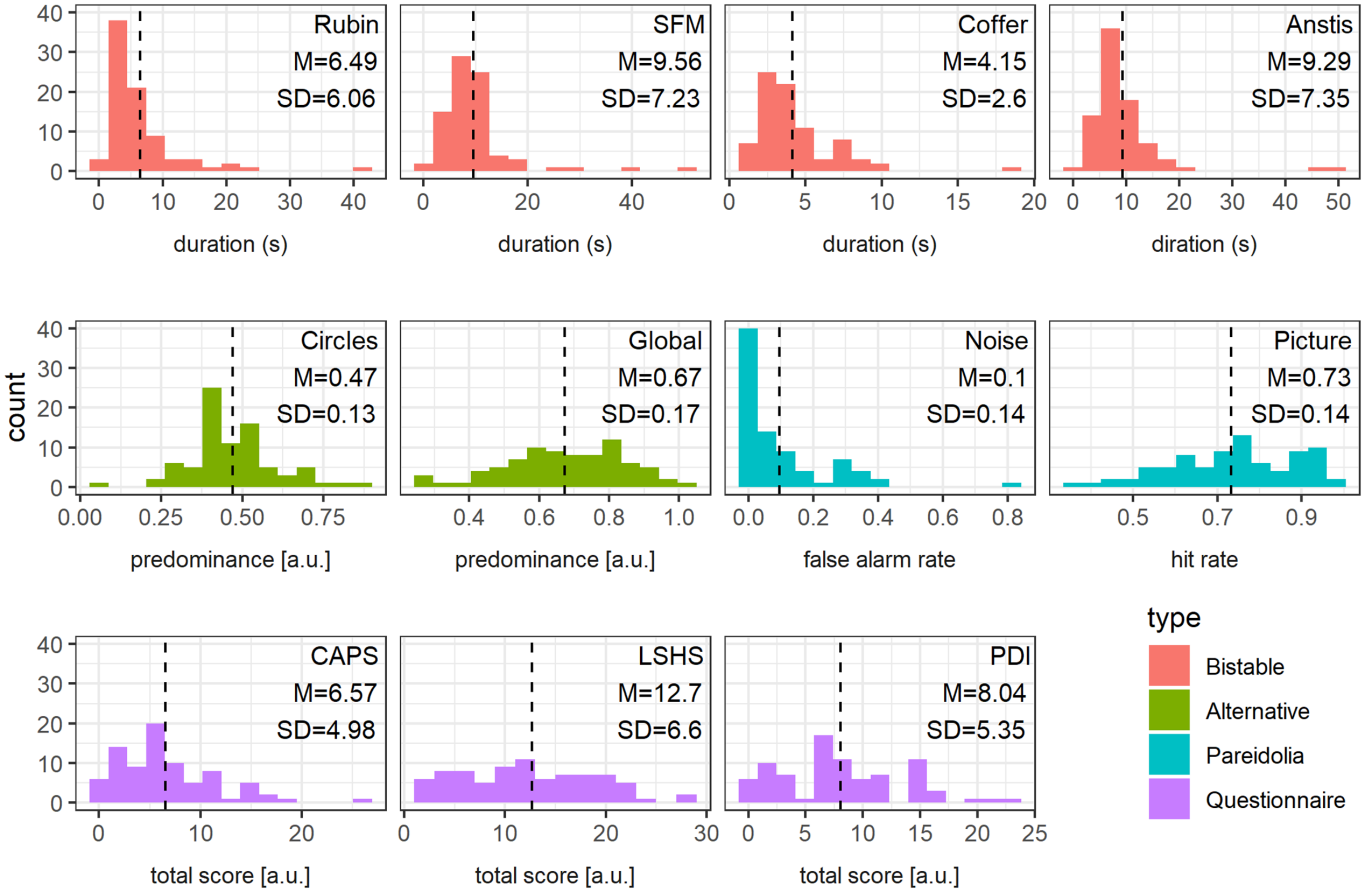
Data distribution. Frequency histogram of each variable together with mean (also indicated as a vertical dashed line) and standard deviation values, color-coded according to the variable type: bistable stimuli (red), alternative percept predominance (green), Pareidolia tasks (blue) and Questionnaires (violet).

Data for the Picture Pareidolia task was missing for two participants for technical reasons. For these two subjects the hit values were replaced by the group average. All remaining datasets were complete. The distributions, as well as means and standard deviations of all measured variables are presented in Figure 4. All but one variable, the false alarm rate in the Noise Pareidolia task, revealed sufficient variability. Despite our expectation and in contrast to a previous study that reported false alarm rates of 38% for letters and 34% for faces in this task (Liu et al., 2014), we observed extremely low false alarm rates, with 30.49% of all subjects having zero false alarms in the hardest task. We nevertheless included this variable into the subsequent PCA. Removing it entirely, or substituting it with the false alarm rate over the entire Noise Pareidolia experiment (i.e., including noise trials from easy and hard blocks) does not substantially change the distribution of variable loadings across the first two PCA components (see Supplementary Figure 2).

**Figure 5:**
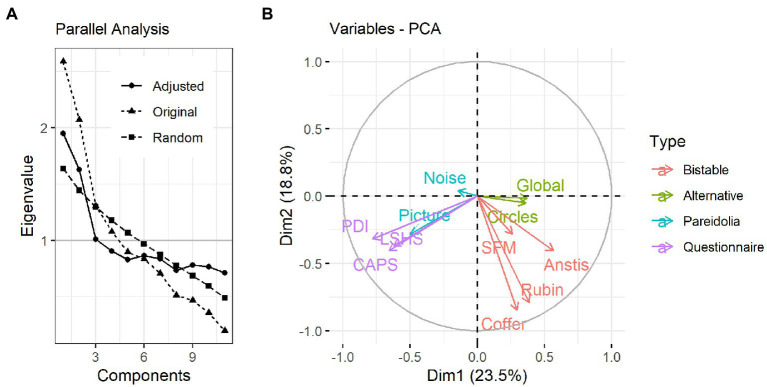
PCA results. **(A)** Parallel analysis result showing the original, random and adjusted eigenvalues for each component. According to the parallel analysis, the first 3 components are the most relevant for explaining variance in the data. **(B)** Biplot showing the distribution of the variables across the first two principal components. Length of an arrow represents its contribution to the respective component, while the angle between each two arrows represents the degree of association between variable pairs (the smaller the angle, the stronger the association).

The outcome of the PCA analysis is shown in Figure 5. Following the parallel analysis (Figure 5A), 3 principal components were kept for subsequent varimax rotation, which together explained 54% of the variance in the data.

Following the rotation, the hit rate in the Picture Pareidolia task as well as the questionnaires showed similar loadings on the first rotated component (Figure 5B), while the dominance durations of all four bistable illusions showed similar loadings onto the second principal component (Table 1). The third component was dominated by the Noise Pareidolia task.

**Table 1:** Variable contribution to the 3 PCA components after varimax rotation.

	RPC1	RPC2	RPC3
Rubin	−0.04	−0.59	−0.07
SFM	0.22	−0.34	0.32
Anstis	0.05	−0.34	−0.39
Coffer	−0.06	−0.63	0.05
Global	0.04	−0.02	−0.41
Circles	0.02	−0.04	−0.4
Noise	0.16	−0.06	0.51
Picture	−0.24	−0.1	0.35
CAPS	−0.56	−0.03	−0.04
LSHS	−0.53	−0.01	−0.05
PDI	−0.52	0.02	0.14

Values represent the loading of each variable onto each of the first three rotated components (correlation between the variable and the extracted component). Please see Data Analysis section 2.3.2 for details.

**Figure 6:**
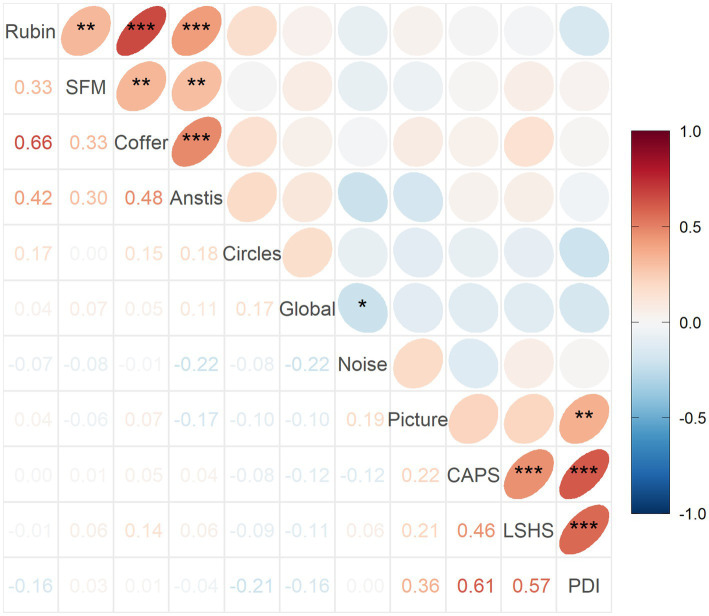
Spearman’s correlation coefficients between variable pairs and their significance. *** *p* < 0.001, **p < 0.01, *p < 0.05. Corresponding Pearson’s results are shown in Supplementary Figure 3.

This pattern is reflected in the correlation structure between variables which is shown in Figure 6. As expected, there are positive moderate to strong correlations between the questionnaires, ranging from 0.46 to 0.61 (*p* < 0.01). There are also positive moderate to strong correlations between the reversal rates of bistable illusions, ranging from 0.30 to 0.66 (*p* < 0.01). Most importantly, there is a moderate positive correlation between the hit rate in Picture Pareidolia task and the delusion score (PDI, *R* = 0.36, *p* < 0.01). Finally, there is no significant correlation of Noise Pareidolia with any other variable. Crucially, there is no positive association between any of the illusion measures on the one hand, and the pareidolia and questionnaire scores on the other hand.

**Figure 7:**
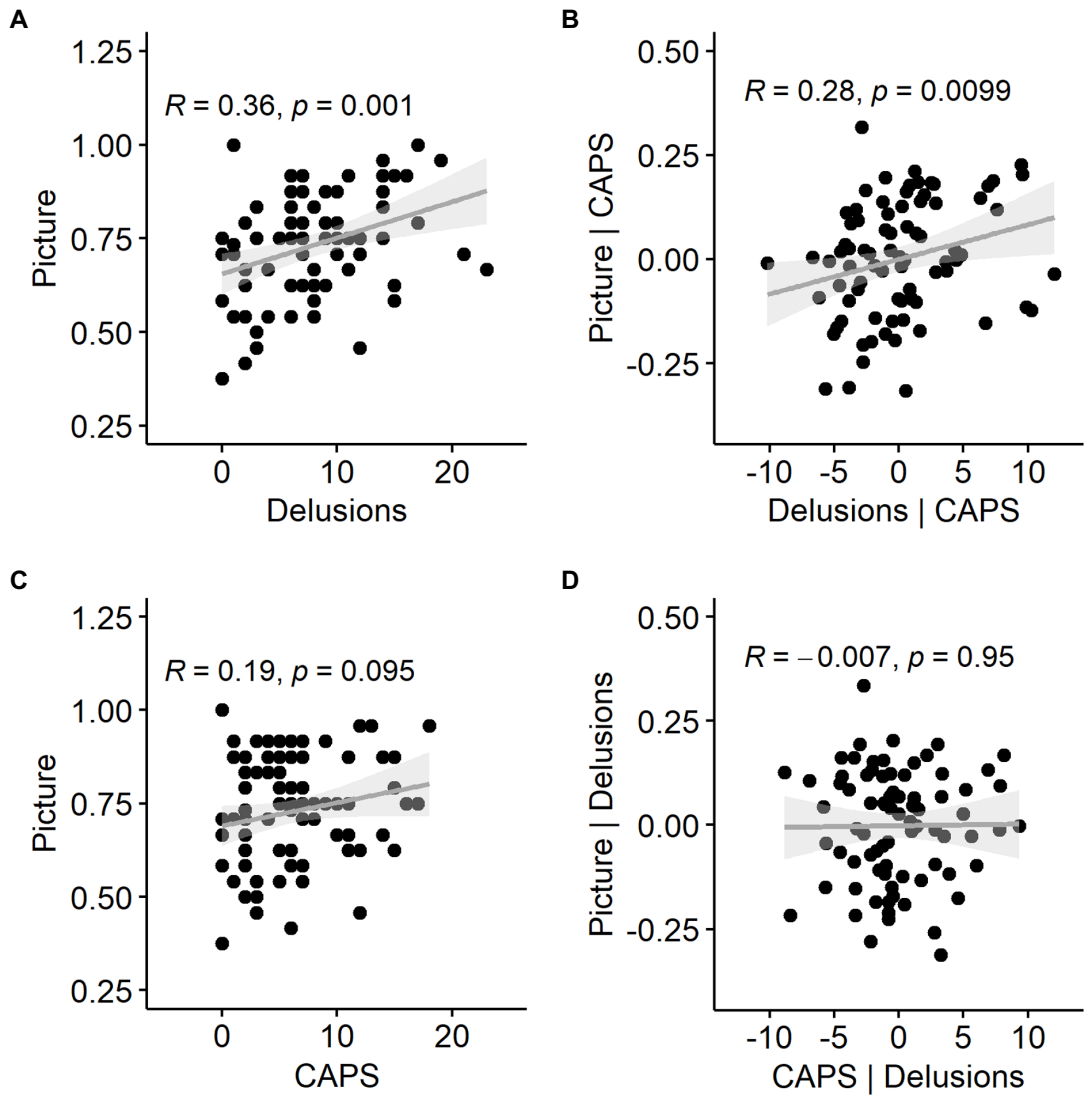
Relationship between pareidolia proneness in the Picture Pareidolia task, tendency for delusional ideation and abnormal perceptual experiences (hallucinations). **(A)** Scatter plot of the delusion score and Picture Pareidolia hit rate with a regression line and Spearman’s correlation coefficient. **(B)** same as **(A)**, but with hallucination-related variance as measured by the CAPS questionnaire removed. **(C)** Relationship between pareidolia proneness and hallucinations as measured by CAPS. **(D)** Same as **(C)**, but with delusion-related variance removed.

Hit rate in the Picture Pareidolia task thus correlated most strongly with the delusion questionnaire (Figure 7A), but delusion scores also correlated with hallucination scores. We therefore additionally wanted to determine the unique contribution of delusion scores to explaining the Picture Pareidolia proneness. We calculated Spearman’s correlation coefficient between Picture Pareidolia hit rate and PDI score with CAPS scores regressed out. This analysis still showed a significant positive correlation (Figure 7B). In contrast, repeating the same procedure for hallucination scores (CAPS, Figure 7C) versus Picture Pareidolia hit rate with delusion scores regressed out did not show a significant association (Figure 7D). Similar results were obtained when using LSHS instead of CAPS as hallucination scores (Supplementary Figure 4). This shows that Picture Pareidolia proneness is best explained by the delusion tendency.

The visual acuity test revealed a visual acuity of-0.13 logMAR (*SD* = 0.21), with only 4 participants exceeding the range of normal visual acuity up to 0.65 logMAR. Neither visual acuity nor stereoacuity showed a significant relationship with Picture Pareidolia hit rate (both *p* ≥ 0.23) and the PDI questionnaire score (both *p* ≥ 0.10).

There have been reports of gender differences in pareidolia perception (Pavlova et al., 2015; Proverbio and Galli, 2016). Although the effect was reported specifically for face pareidolia and may be related to women’s general superiority in processing facial information (Zhou and Meng, 2020), given that our sample contained more female than male participants we tested for potential gender differences in our main dependent variables. Both the Picture Pareidolia task and the PDI score revealed no statistically significant gender differences (Picture: *t*(80) = 0.13, *p* = 0.90; PDI: *t*(80) = −0.22, *p* = 0.83).

## 4. Discussion

In the current study we investigated the connection between different visual and non-visual phenomena explained by the predictive coding theory by examining the individual differences in their parameters in healthy young adults. Our results revealed a known relationship between different types of bistable stimuli, but also a previously unreported link between the pareidolia proneness and the tendency for delusional ideation. Crucially, there was no correlation between bistable perception and the remaining phenomena. Our data do not support the hypothesized link between different visual phenomena and suggest instead that they are governed by independent mechanisms. They further suggest that illusory perception in pareidolia is driven primarily by higher-level factors related to thought than by sensory processing that drives bistable illusions.

### 4.1. No common factor for different types of illusory perception

Our study follows the line of research taken previously by other groups for finding common factors that may underlie potentially related perceptual phenomena. In one such study, Cappe et al. (2014) tested whether there is a common factor in visual perception akin to the *g* factor in intelligence. The authors analyzed correlations between a range of basic visual perception tasks such as visual acuity, backward masking, detection and discrimination, finding surprisingly few significant relationships between different aspects of visual perception. Such independence was also reported for different types of visual illusions (Cretenoud et al., 2019), and even between the different examples of the same visual illusion type (Cao et al., 2018). In the study by Cao et al. (2018), the authors examined a potential relationship between the alternation rates in different types of bistable stimuli, concluding that the degree of association varied from very weak to moderate and depended on the extent to which the stimuli engage similar perceptual mechanisms. We also observed weak to moderate correlations between dominance durations of different bistable stimuli in our study, with only one strong correlation between the Face-Vase illusion and the Coffer illusion, possibly because both involve figure-ground segregation mechanisms. Importantly, we observed no positive association between bistable illusions and pareidolias, which we hypothesized are both driven by perceptual top-down mechanisms. This and similar studies thus demonstrate that visual perception is less homogeneous than one would expect, and that even the apparently similar visual phenomena may result from entirely different perceptual, and potentially also neural, processes.

### 4.2. Hierarchical nature of predictive coding

Overall, our main finding of independent variation in bistable perception properties on the one hand, and pareidolia proneness and delusion tendency on the other hand is broadly consistent with the idea that top-down mechanisms determine perception at different hierarchical levels, and that these levels may be largely independent from each other (Sterzer et al., 2018). And yet several specific results of our study contradict previous findings reported in the field. For example, one previous study could show a *negative* correlation between delusional ideation and perceptual stability of bistable stimuli and a *positive* correlation between delusional ideation and belief-induced perceptual bias in a group of healthy individuals, suggesting an opposite role of weak sensory and strong cognitive priors for the emergence of delusions (Schmack et al., 2013). We observed neither of the two relationships in our data. The absence of a negative relationship between perceptual stability and delusion tendency as measured with PDI scores could partially be explained by the specific measure used to quantify perceptual stability. In the study of Schmack et al., an intermittent stimulus presentation paradigm, in which a bistable stimulus is regularly removed and then shown again, was used to calculate “survival probability,” i.e., the likelihood of the perceptual interpretation remaining the same across the stimulus removal periods. In the current study, we used a more classical bistable perception paradigm and measured perceptual stability as an average dominance duration. Average dominance duration is directly related to survival probability, as it measures the duration of perceptual stability before a spontaneous destabilization and a switch to the alternative percept occurs. The two measures appear to show a strong correlation across individuals (Figure 2; Leopold et al., 2002), and were shown to be driven by the same oscillatory mechanisms (Zhu et al., 2022). Nevertheless, we cannot exclude the possibility that survival probability in intermittent stimulus presentation may be a measure that better captures the perceptual stability mechanisms. Given the relatively weak association between bistable perception and PDI, even minor deviations in how perceptual stability is quantified could make a difference. Our data also show that correlations between perceptual stability measures derived from different bistable stimuli is moderate at best. It therefore remains an open question to which degree an association between PDI and survival probability in structure-from-motion illusion would generalize to other bistable stimuli.

Interestingly though, we found a weak negative correlation between the PDI score and the tendency for an alternative percept in the Anstis and Coffer illusions (and also for the dominance duration of the Anstis illusion, which is likely to be driven by a typically longer global percept). These correlations, although not reaching significance in our slightly smaller sample than that of Schmack et al., are similar in magnitude (for a comparison, see Pearson’s correlation coefficients reported in Supplementary Figure 3). They do speak in favor of the hypothesis that delusional tendencies are related to weak top-down sensory predictions if the latter are expressed as the alternative percept predominance.

### 4.3. What drives pareidolia proneness

The key finding of our study is a relationship between the delusion tendency and pareidolia proneness as measured in the Picture Pareidolia task. Delusions are complex cognitive phenomena that involve interpretation, attribution, reasoning and causal inference. This relationship indicates that detecting pareidolias in natural scenes is not limited to perception and object recognition, but involves complex higher-level functions related to cognition and thought.

Our results complement findings of a previous study reporting that pareidolia proneness is related to hallucination tendency in healthy individuals (Smailes et al., 2020). In the current study, where we examined both, hallucination and delusion tendency, we could show that the association with delusions is stronger and may be the actual primary driver behind the association with hallucinations. Interestingly, the same study measured schizotypy in healthy adults and could not find evidence for the latter contributing to the pareidolia-hallucination association (Smailes et al., 2020). Together with previous findings, our results suggest that the association between pareidolia and delusion tendency is not a reflection of general psychotic tendency of an individual, but is confined to its one specific manifestation, namely delusions. It follows that increased pareidolia proneness previously reported in schizophrenia patients (Abo Hamza et al., 2021) may be explained solely by the core symptom of delusions.

### 4.4. Picture and noise pareidolia

An interesting aspect of our findings is that we observed effects only in the Picture Pareidolia task, but not in the Noise Pareidolia task. One rather technical reason for this could be the skewed distribution of false alarm rates for the Noise Pareidolia task in our sample, which could have led to floor effects (see Figure 4). However, this must have also been the case in the study of Smailes et al., (2020) (false alarm rate of 0.12 in Smailes et al., (2020) versus 0.10 in this study), which nevertheless could report an association between a similar Noise Pareidolia task and hallucinations. More recently, a large-scale study with more than 1,000 participants revealed that the effect size for the association between hallucinatory experiences and false alarm rates in a detection task is rather small (Spearman’s r = 0.14), even when the average false alarm rate is sufficiently high (Moseley et al., 2021). Effects of this size would not have been possible to detect with our sample size, which could be another reason for this negative finding.

Another, more substantial reason could be the different aspects of false perception that are measured in the Picture Pareidolia and the Noise Pareidolia tasks. The Noise Pareidolia task, which is also referred to as “reality discrimination” task, requires a high level of uncertainty about the sensory input and a suggestive instruction that induces expectations of a specific stimulus (Salge et al., 2021). Furthermore, the usage of Noise Pareidolia-type tasks for quantifying hallucinatory tendencies in general population requires high metacognitive confidence ratings that a participant saw something. False alarms under low confidence can be attributed to, e.g., a more liberal reporting criterion or social pressure of experimental situation (Salge et al., 2021). In contrast, the Picture Pareidolia task contains clear sensory input and less expectation to detect a specific stimulus (i.e., weaker sensory priors). It rather represents an individual’s tendency to re-interpret or even overinterpret the existing input in another meaningful way. This aspect actually relates pareidolias in everyday life and in natural images to delusional ideations, which are thought to result from weak sensory priors which enable higher-level aberrant interpretation. While we provide the correlational evidence for this relationship in healthy individuals, further studies are needed to test whether it holds in, e.g., clinical samples.

Since the low false alarm rate in the Noise Pareidolia task in our study deviates significantly from the original report by Liu et al. (2014), which served as the basis for our paradigm, we would like to briefly discuss potential reasons for this discrepancy. In the hardest task block of Liu et al. (2014) participants were instructed that 50% of all images will contain the target item (face or letter depending on the block), thereby creating a strong expectation in participants that some images will contain targets. We slightly modified this instruction, only informing participants that in this block target items will be very hard to detect (i.e., without specifying the exact percentage of target-present trials). Our modification was aimed at reducing potential effects of socially desirable behavior in our participants, who would otherwise feel the pressure to report targets even though they do not perceive them. More technical factors, like monitor properties or surrounding experimental conditions, may have also played a role. Low false alarm rates were apparent to us in the pilot testing, and we intentionally reduced the duration of stimulus presentation compared to Liu et al. (2014) to make the stimulus processing harder for the participants, but this modifications appeared to be insufficient.

### 4.5. Limitations

In this final section of our manuscript we would like to address several limitations of our study. First, as discussed above, we observed low false alarm rates in the Noise Pareidolia task, which could have precluded finding significant associations with other dependent variables due to potential floor effects. Therefore, future studies using similar tasks should put more effort into careful calibration of experimental parameters and/or instruction to achieve higher false alarm rates. Second, a prevalence of women over men in our participant sample may have caused a potential bias if, for example, a certain dependent variable is more pronounced in individuals of one gender than of the other. We could rule out such biases for the significant associations by testing for the effects of gender post-hoc, but it remains unclear whether gender disbalance could have caused null effects for some associations. Therefore, future studies should also pay more attention to recruiting a gender-balanced participant sample. Finally, the results we report here are based on a sample of healthy individuals. It is not clear to what extent anomalous perception or delusional ideations reported by our participants are qualitatively similar to hallucinations and delusions that are encountered as symptoms in clinical populations. Future studies can be aimed at testing the relationship between pareidolia and delusional ideations in clinical populations with delusions, such as schizophrenia patients.

## 5. Conclusion

Overall, our results speak against a common mechanism behind different perceptual and non-perceptual phenomena explained by the predictive coding theory. However, they are consistent with the notion of the hierarchical predictive processing and suggest that lower-level perceptual and higher-level cognitive predictions operate independently. They also place the phenomenon of pareidolia at the higher cognitive level of the prediction hierarchy.

## Data availability statement

The datasets presented in this study can be found in online repositories. The names of the repository/repositories and accession number(s) can be found at: Open Science Framework (OSF) https://osf.io/jnz4e/.

## Ethics statement

The studies involving human participants were reviewed and approved by Ethics Committee of the University of Graz. The patients/participants provided their written informed consent to participate in this study.

## Author contributions

ML: conceptualization, software, investigation, formal analysis, writing – original draft, writing – review and editing, and project administration. AI: validation, resources, supervision, and writing – review and editing. BH: conceptualization, software, and writing – review and editing. NZ: conceptualization, methodology, software, formal analysis, writing – original draft, visualization, supervision, and funding acquisition. All authors contributed to the article and approved the submitted version.

## Funding

This work was funded by the BioTechMed-Graz, Austria (Young Research Group Grant to NZ) and by the University of Graz.

## Conflict of interest

The authors declare that the research was conducted in the absence of any commercial or financial relationships that could be construed as a potential conflict of interest.

## Publisher’s note

All claims expressed in this article are solely those of the authors and do not necessarily represent those of their affiliated organizations, or those of the publisher, the editors and the reviewers. Any product that may be evaluated in this article, or claim that may be made by its manufacturer, is not guaranteed or endorsed by the publisher.

## Footnotes

^1^ http://www.neuro-o.se/CritVis/cVis2.html#3DV

^2^ www.limesurvey.org

## Supplementary material

The Supplementary material for this article can be found online at: https://www.frontiersin.org/articles/10.3389/fpsyg.2022. 1067985/full#supplementary-material
