# Supplementary material for: No common factor for illusory percepts, but a link between pareidolia and delusion tendency: A test of predictive coding theory
Source: Front Psychol. 2023 Jan 4;13:1067985. doi: 10.3389/fpsyg.2022.1067985 (PMC9928206; doi:10.3389/fpsyg.2022.1067985)
Supplement: Supplementary file 1 [file Data_Sheet_1.pdf]

Supplementary Material

1 Supplementary Figures and Tables

1.1 Supplementary Figures

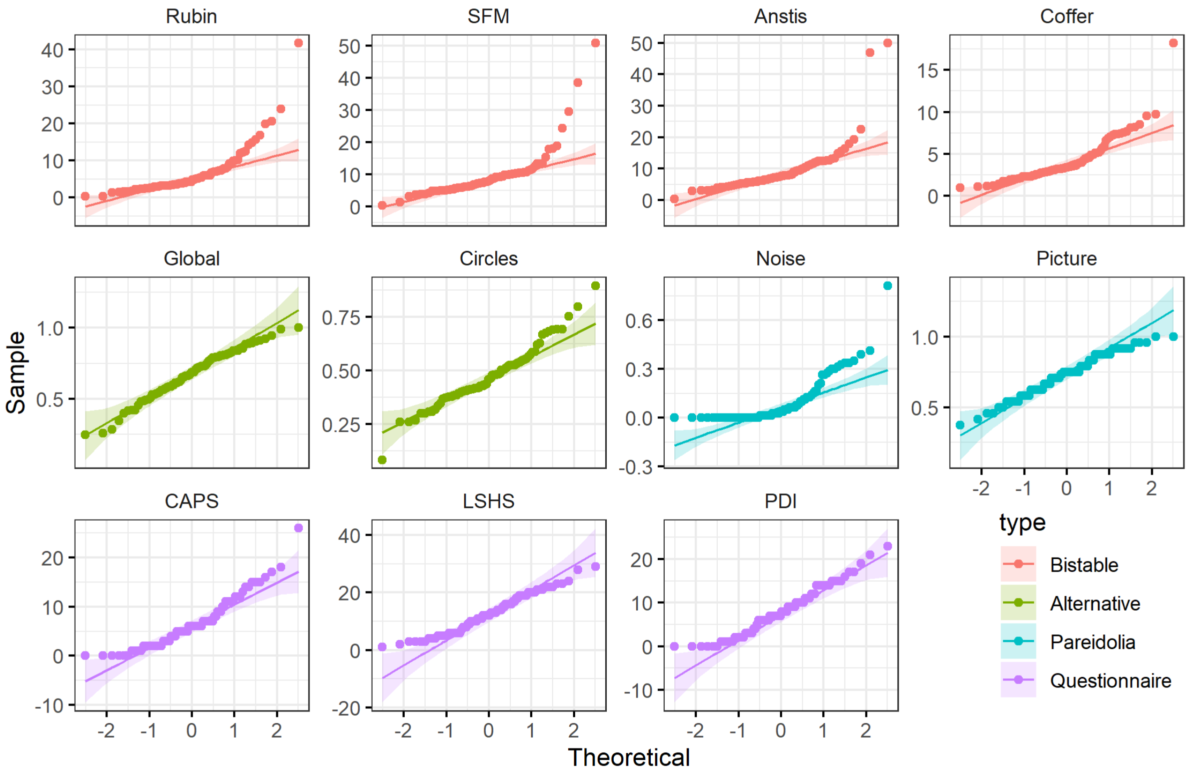

Supplementary Figure 1. Q-Q plots demonstrating the deviation of each variable's distribution from the normal distribution

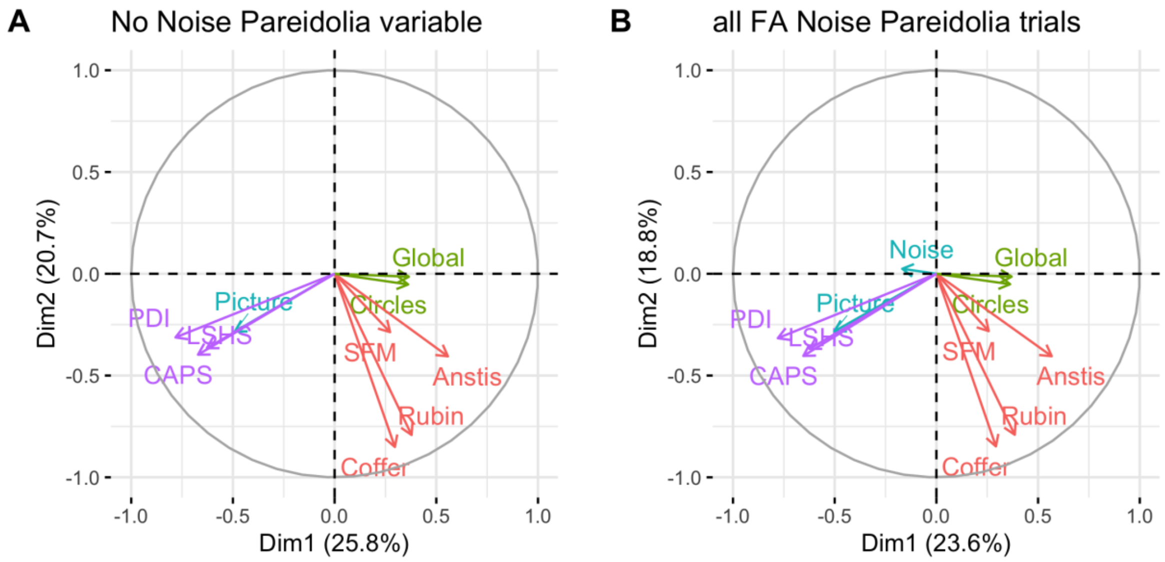

Supplementary Figure 2. Same analysis as shown in Figure 5, but with Noise Pareidolia variable removed **(A)** or with Noise Pareidolia False alarms calculated over the entire experiment rather than in the pure noise blocks only **(B)**.

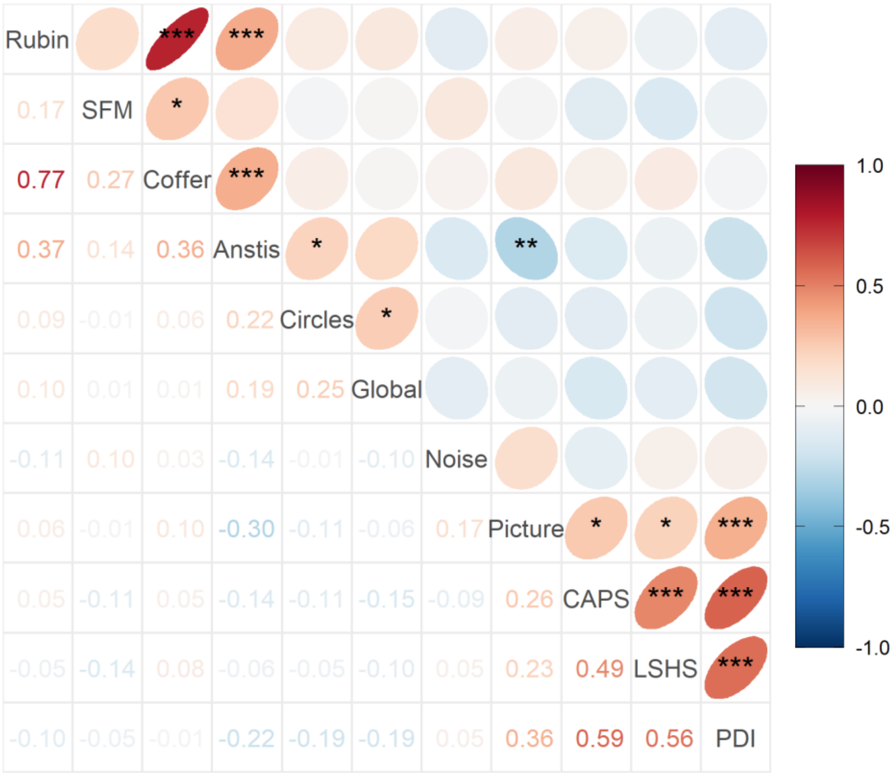

Supplementary Figure 3. Same as Figure 6, but with Pearson's correlation coefficients reported instead of Spearman's for a more straightforward comparison with previous studies.

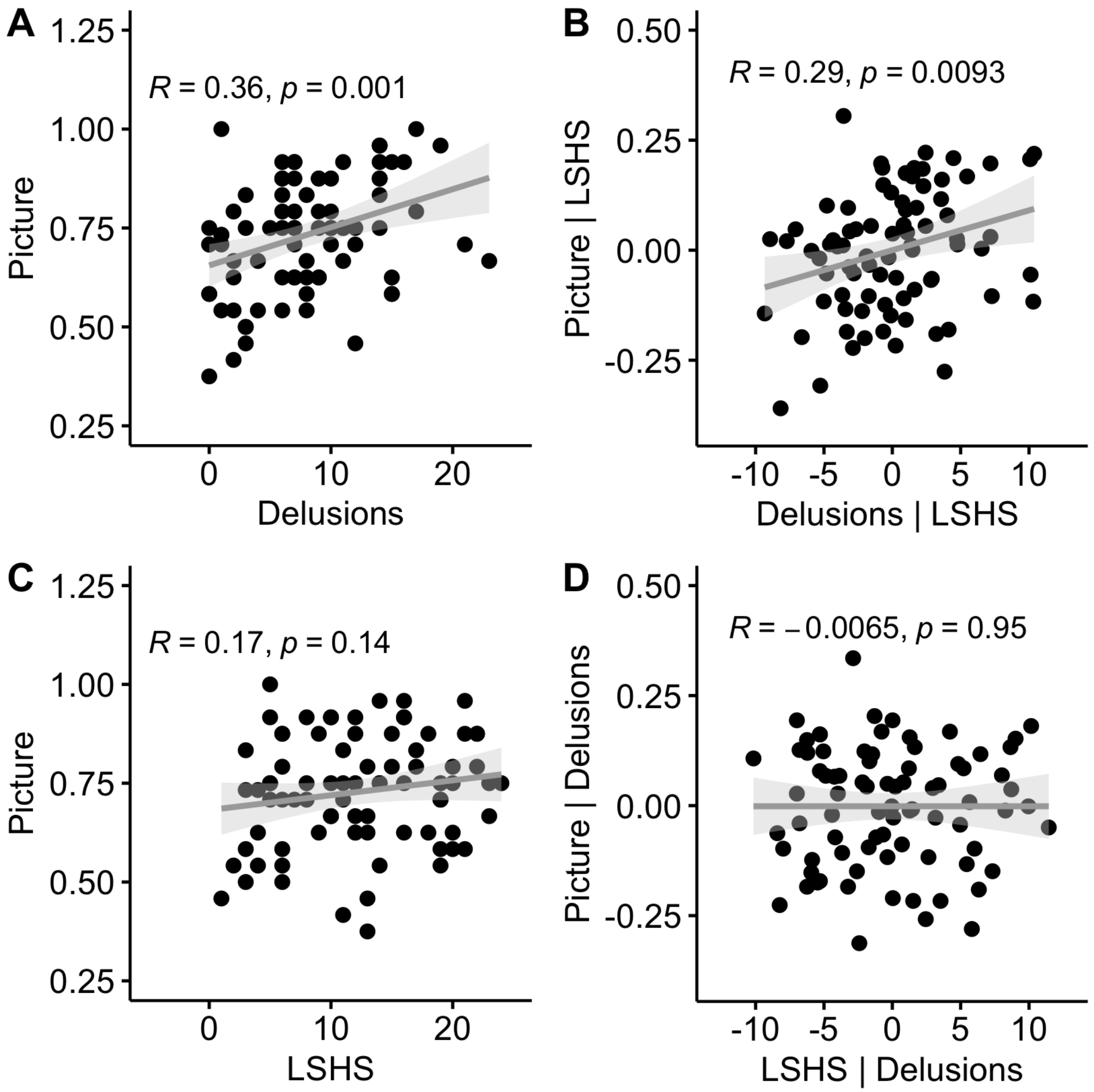

Supplementary Figure 4. Same as the main Figure 7, but with LSHS instead of CAPS as hallucination questionnaire.

1.2 Supplementary Tables

	**CAPS** (Bell et al., 2006)	**LSHS-R** (Bentall and Slade, 1985; Lincoln et al., 2009)	**PDI** (Peters et al., 1999; Lincoln et al., 2009)
Internal consistency (Cronbach's alpha)	*0.87	*0.79<br><br>0.88	*0.88<br><br>0.85
Test-retest reliability (Pearson's R)	*0.77	*0.84	*0.82
Mean	*7.3	7.0	7.0
SD	*5.8	6.9	5.5

Supplementary Table 1. Questionnaire information summary. Internal consistency, test-retest reliability, mean and standard deviation of the measured sample (healthy individuals) for the CAPS, LSHS and PDI. *Asterisk indicates that the value is provided for the English test version.

2 Supplementary References

Bell, V., Halligan, P. W., and Ellis, H. D. (2006). The Cardiff Anomalous Perceptions Scale (CAPS): a new validated measure of anomalous perceptual experience. *Schizophr. Bull.* 32, 366–77. doi: 10.1093/schbul/sbj014.

Bentall, R. P., and Slade, P. D. (1985). Reality testing and auditory hallucinations: a signal detection analysis. *Br. J. Clin. Psychol.* 24 (Pt 3), 159–69. doi: 10.1111/j.2044-8260.1985.tb01331.x.

Lincoln, T. M., Keller, E., and Rief, W. (2009). Die Erfassung von Wahn und Halluzinationen in der Normalbevölkerung. *Diagnostica* 55, 29–40. doi: 10.1026/0012-1924.55.1.29.

Peters, E. R., Joseph, S. A., and Garety, P. A. (1999). Measurement of delusional ideation in the normal population: introducing the PDI (Peters et al. Delusions Inventory). *Schizophr. Bull.* 25, 553–76. doi: 10.1093/oxfordjournals.schbul.a033401.
